# **IL-6 trans-signaling: an overlooked driver of retinal neovascularization?**

**DOI:** 10.1007/s10456-025-10022-8

**Published:** 2025-12-19

**Authors:** Malte Jung, Jan N. Ness, Melanie E. Schwämmle, Julian Rapp, Stefaniya Boneva, Olaf Groß, Julia Mitschke, Günther Schlunck, Hansjürgen Agostini, Luciana Hannibal, Felicitas Bucher

**Affiliations:** 1 Eye Center, Medical Center - Faculty of Medicine, University of Freiburg, 79106 Freiburg, Germany; 2 Institute of Pharmaceutical Sciences, Faculty of Chemistry and Pharmacy, University of Freiburg, 79104 Freiburg, Germany; 3 Faculty of Biology, University of Freiburg, 79104 Freiburg, Germany; 4 Department of Medicine I, Medical Center - Faculty of Medicine, University of Freiburg, 79106 Freiburg, Germany; 5 CIBSS - Centre for Integrative Biological Signaling Studies, University of Freiburg, 79104 Freiburg, Germany; 6 Institute of Neuropathology, Medical Center - Faculty of Medicine, University of Freiburg, 79106 Freiburg, Germany; 7 Center for Basics in NeuroModulation (NeuroModulBasics), Faculty of Medicine, University of Freiburg, 79106 Freiburg, Germany; 8 Institute of Molecular Medicine and Cell Research, Faculty of Medicine, University of Freiburg, 79104 Freiburg, Germany; 9 Laboratory of Clinical Biochemistry and Metabolism, Department of General Pediatrics, Adolescent Medicine and Neonatology, Medical Center - Faculty of Medicine, University of Freiburg, 79106 Freiburg, Germany

**Keywords:** IL-6 signaling, Vitreoretinal vascular disease, Angiogenesis, Metabolism, Endothelial barrier disruption

## Abstract

**Background:**

Vascular endothelial growth factor (VEGF) is regarded as the essential promoter of vitreoretinal vascular diseases that threaten eyesight, such as proliferative diabetic retinopathy (PDR). Therefore, VEGF is the primary therapeutic target in these diseases, but not all patients respond adequately to VEGF inhibition. This raises the question if other factors contribute to disease modulation. PDR evolves in an interplay of pathological processes including inflammation, barrier integrity loss, aberrant angiogenesis, and metabolic dysregulation. Interleukin-6 (IL-6), recognized for its pro-inflammatory properties, was the focus of this study.

**Aim:**

Investigate IL-6 mediated angiogenic potential and disease-relevant mechanisms in the context of VEGF driven vitreoretinal disorder.

**Methods:**

Levels of IL-6 and soluble IL-6 receptor (sIL-6R) were quantified in patient samples using ELISA. In vitro, the functional effect and downstream signaling patterns of IL-6, sIL-6R and VEGF on vascular endothelial cells were analyzed with western blot, spheroid sprouting-, migration-, seahorse assays and LC–MS/MS.

**Results:**

Vitreous samples from PDR patients showed elevated levels of IL-6 and its corresponding soluble IL-6 receptor (sIL-6R) compared to clinical control groups. In vitro, IL-6 trans-signaling (IL-6 + sIL-6R) leads to a pro angiogenic phenotype in human vascular endothelial cells demonstrated in migration and spheroid sprouting assays, mirroring the effects of VEGF. Interestingly, IL-6 trans- and VEGF-signaling differ in their effects on barrier integrity and metabolic profile. IL-6 trans-signaling disrupts endothelial barrier function and shows an increased mitochondrial oxygen consumption rate in the Seahorse assay, as well as lower produced lactate levels compared to VEGF. Tocilizumab, an IL-6R antibody, showed additive treatment effects to anti-VEGF therapeutics regarding angiogenesis and VEGF induced metabolic drive in vitro.

**Conclusion:**

IL-6 trans-signaling functions as an independent promoter of vitreoretinal vascular disease and therapeutic targeting of its pathway could beneficially complement current anti-VEGF treatment protocols.

**Supplementary Information:**

The online version contains supplementary material available at 10.1007/s10456-025-10022-8.

## Introduction

With estimated 700 million people suffering from diabetes by 2045 and about 35% of these patients affected by diabetic retinopathy (DR), DR represents a major current and upcoming global challenge [1, 2]. So far, vascular endothelial growth factor (VEGF) appears to be the main driver for the progression of non proliferative-DR to proliferative-DR (PDR) characterized by ischemia leading to aberrant neovascularization, blood vessel leakage and macular edema (DME) [3–5]. Therefore, intraocular injection of anti-VEGF drugs is an attractive therapeutic approach for patients with PDR and DME. However, ceiling effects with respect to gain in visual acuity, non-responders to anti-VEGF treatment [6] and a short biological drug half-life period requiring frequent injections, constitute a burden that limits therapeutic success [7]. Regarding the multifaceted pathogenesis of DR which includes inflammation [8], neurodegeneration [9] and dysregulated metabolism [10], other cytokines than VEGF should also be considered as potential disease-modifying factors [11]. In this study, we evaluate the potential of the IL-6 signaling pathway as complementary therapeutic target for the treatment of PDR and DME to enhance existing anti-VEGF therapy.

To this end, it is important to first understand the complex IL-6 signaling cascade. IL-6 is part of the IL-6 cytokine family commonly signaling through the receptor subunit glycoprotein 130 kDa (gp130) [12]. IL-6 interacts with gp130 through two distinct pathways: classical IL-6- (cis) signaling and trans-signaling. Cis-signaling involves two molecules of IL-6 binding to two corresponding trans-membrane bound IL-6 receptors (IL-6R) and two gp130 receptor subunits. The heterohexameric signaling complex including the homodimerization of gp130 recruits and activates Janus kinases (JAKs) which in turn phosphorylate signal transducer and activator of transcription protein 3 (STAT3). This pathway is limited to cells endogenously expressing IL-6R including certain leukocyte subgroups, hepatocytes, epithelial and vascular as well as retinal microvascular endothelial cells [13– 17].

IL-6 trans-signaling on the other hand is available also to cells not expressing IL-6R. Soluble IL-6R (sIL-6R), mainly generated by proteolytic cleavage, binds to IL-6 in the extracellular space and then interacts with gp130, again leading to a heterohexameric signaling complex. While differences between IL-6 cis- and trans-signaling are still largely unexplored at the molecular level, functional effects of IL-6 are well characterized. IL-6 trans-signaling is described to induce pro-inflammatory activities, whereas IL-6 cis-signaling via the membrane bound IL-6R affords protection and regeneration [18, 19].

The IL-6/JAK/STAT3 pathway with its distinctive roles of cis- and trans-signaling is recognized as central signaling pathway and therapeutic target in the field of rheumatoid arthritis, colitis ulcerosa and tumor angiogenesis [20]. With regards to human vitreoretinal diseases, it has repeatedly been shown that IL-6 levels are significantly increased in the vitreous of PDR patients [21–23]. Interestingly, patients with elevated aqueous humor IL-6 levels have poorer visual acuity under anti-VEGF treatment for DME or neovascular age-related macular degeneration [24]. Targeting the IL-6 signaling cascade is gaining ground in ophthalmology and is under investigation for its benefits in treating uveitic macular edema [25].The role of sIL-6R, the pivotal molecular part of IL-6 trans-signaling, however remains largely unexplored. So far, only one clinical study looked at sIL-6R levels in the vitreous of PDR patients finding them to be elevated [26]. Preclinical studies support that IL-6 trans-signaling causes inflammation and endothelial barrier dysfunction in vascular endothelial cells in the retina as well [27] and that trans-signaling may be more potent than classical cis-signaling [28]. However, the effect of IL-6 cis- and trans-signaling in the context of VEGF driven disease and its potential as complementary therapeutic target to VEGF remains elusive. To better elucidate the impact of IL-6 signaling on vitreoretinal diseases we first examined IL-6 cis- and trans-signaling on different disease-driving aspects including angiogenesis, endothelial barrier integrity and cell metabolism particularly in the framework of VEGF activity to mimic disease conditions in vitro. We then used tocilizumab, a well-established humanized monoclonal anti-IL-6R antibody used in treating rheumatoid arthritis [29], to test for enhanced treatment effects in combination with a VEGF inhibitor.

The aim of our study was to investigate the functional effects of IL-6 cis- and trans-signaling signaling and its interplay with VEGF, mimicking disease conditions. We provide strong evidence that IL-6 trans-signaling contributes to disease specific pathological processes such as angiogenesis, barrier function and displays distinct differences in metabolic energy supply pathways compared to VEGF. Combined targeting of the VEGF and IL6-signaling cascades offers a promising avenue for improved therapeutic strategies.

## Methods

### Patient sample acquisition

Undiluted human vitreous from patients with PDR (*n* = 17) as primary disease group were collected during pars plana vitrectomy. In the same manner we collected samples of patients with macular pucker (*n* = 17) or macular hole (*n* = 19) as clinical control groups. Matching patient blood samples were collected additionally to evaluate systemic IL-6 and sIL-6R levels. We excluded patients with the diagnosis of diabetes mellitus from our macular pucker (MP) and macular hole (MH) control cohorts. Each patient underwent vitrectomy as part of standard care. Prior to surgery, written informed consent was obtained from the patients for the use of biological samples for research. This study was approved by the local Ethics Committee of the Albert-Ludwigs-University Freiburg (EK 17/17, EK 20-1165). After collection samples were centrifuged at 4 °C, vitreous at 500 x g for 20 min and blood at 3000 x g for 15 min. The resulting supernatant was aliquoted and frozen at −80 °C until further usage. Only measurements within the detection range of the assays were considered.

### Cytokine quantification via ELISA

Cytokine levels were detected using the High Sensitivity IL-6 Human ELISA Kit (#BMS213-2HS, Thermo Fisher Scientific) and the IL-6 receptor (soluble) human ELISA kit (#BMS214, Thermo Fisher Scientific).

### Cell culture

All in vitro experiments were performed using human umbilical vein endothelial cells (HUVECs, #2519A; Lonza). HUVECs were used up to passage 6 and cultured in Endothelial Growth Medium-2 (EGM, #CC-3162; Lonza). Prior to experiments, HUVECs were transferred to Endothelial Basal Medium-2 (EBM, #CC-3156; Lonza) containing 2% fetal bovine serum (FBS, S0615, Biochrom, Berlin, Germany). If not stated otherwise the latter condition was used for negative controls (EBM). The following cytokines and inhibitors were used for treatment experiments: VEGF (25ng/mL, #100-20; Peprotech), human IL-6 (100ng/mL, 7270-IL, R&D Systems) and human soluble IL-6 receptor (200ng/mL, 227-SR, R&D Systems), Aflibercept (50pg/ml, Eylea, Bayer GmbH) and Tocilizumab (100 µg/ml, RoActemra, Roche).

### Western blot

15 min after cytokine treatment HUVECs were lysed with T-Per buffer (#78510; Thermo Fisher Scientific) including 1% of protease inhibitor (#87786; Thermo Fisher Scientific) and 1% of phosphatase inhibitor (#78420, Thermo Fisher Scientific).

Denatured protein samples were separated by gel electrophoresis, transferred to an Immobilon-P PVDF Membrane (#IPVH00010, Merck Millipore). The membrane was then blocked with 3% of bovine serum albumin (BSA) (#11926.03, Serva) followed by incubation with primary antibodies overnight at 4 °C: pSTAT3 (Tyr705) rabbit mAb (#9145, Cell Signaling Technology), pSTAT3 (Ser727) rabbit mAb (#9134, Cell Signaling Technology), pERK p44/42 MAPK (T202/Y204) rabbit mAb (#4370, Cell Signaling Technology), pAkt (Ser473) rabbit mAb (#9271, Cell Signaling Technology), mouse GAPDH Ab (#MAB374, Merck). Membranes were then incubated with the secondary antibodies for 1 h the following day (Anti-rabbit: #115-035-003, Anti- mouse: #111-035-003, Jackson ImmunoResearch). Western blot signals were developed with the ECL Prime Western Blotting System (#RPN2232, GE Healthcare) and images were collected using a Fusion FX system (Vilber, Collégien, France).

### Spheroid sprouting assay

The assay was performed as published earlier [30]. In brief, HUVEC spheroids were formed in hanging drops and later seeded into a 3-dimensional collagen matrix (Collagen I, Rat Tail Cat; #354236, Corning) and treated with different combinations of VEGF, IL-6, sIL-6R, Aflibercept and Tocilizumab overnight (15 h). Images were taken and the total sprouting length was quantified by using the measuring tool of ImageJ-Fiji [31]. For comparison, each treatment was normalized to the control group to calculate the relative sprouting length.

### Scratch-wound-assay

20.000 HUVECs per well were seeded into a 96-well IncuCyte ImageLock plate (#BA-04856, Sartorius). Following overnight starvation (15 h) in EBM containing 2% fetal bovine serum (FBS, S0615, Biochrom, Berlin, Germany), scratching was performed using the IncuCyte WoundMaker (IncuCyte Cell Migration Kit, #4493, Sartorius). Directly after specific cytokine treatment cells were imaged every hour for 14 h by the IncuCyte S3 Live-Cell Analysis System (#4647, Sartorius). These images were further analyzed using the integrated IncuCyte software (Integrated Cell Migration analysis module, #9600-0012, Sartorius) to calculate the relative wound density (RWD).

### Immunocytochemistry

HUVECs seeded on coverslips were fixed with 2% PFA for 20 min (Paraformaldehyd, #0335.1, Carl Roth GmbH & Co. KG, Karlsruhe, Germany) after incubation for 12 h with the labeled treatment. Coverslips were then blocked with 5% goat serum (Normal Goat Serum, #005-000-121, Jackson ImmunoResearch Laboratories, Ely, U.K.). Samples were incubated with the following primary and secondary antibodies overnight: rabbit anti-human polyclonal Anti-ZO-1 (Zonula Occludens 1, #40-2200, ThermoFisher Scientific Inc.), Phalloidin (Phalloidin-Fluoresceinisothiocyanate, #P5282, Sigma-Aldrich), Occludin monoclonal antibody (#33-1500, Invitrogen), Alexa Fluor^®^ 647 AffiniPure Fab Fragment Goat Anti-Rabbit (#111-607-008, Jackson ImmunoResearch Laboratories). Samples were mounted with Antifade Mountant with NucBlue™ (ProLong™ Glass Antifade Mountant with NucBlue™, #P36981, ThermoFisher Scientific Inc.) and imaged with the Leica Confocal microscope (Leica TCS SP8, Leica microsystems).

### RTCA xcelligence

HUVECs were seeded on E-Plate 16 (#300600890, Agilent Technologies, Inc., U.S.A.) and cultivated until constant impedance was reached. Medium was changed every day. 24 h before treatment cells were starved in EBM with 2% FBS. Before treatment impedance was measured every hour, after treatment impedance was measured every 5 min. Results were presented as Cell Index ${\text{CI}}\;({\text{Cell}}\;{\text{Index}})=\frac{{impedance\;({t_n}) - (tabscence\;of\;cells)}}{{nominal\;impedence\;value}}$. For impedance measurements the XCELLigence RTCA DP (Agilent Technologies, Inc., U.S.A.) was used.

### Extracellular metabolic flux analysis

Real-time shifts in cellular bioenergetics were detected by using a Seahorse XFe96-Analyzer (Agilent Technologies), measuring the oxygen consumption rate (OCR) following our previously published workflow (Rapp et al. 2022). HUVECs were pretreated with cytokines (VEGF, IL-6 and sIL-6R) and targeted therapeutics (Aflibercept, Tocilizumab) for 15 h, following a medium change consisting of DMEM (#D5030, Sigma-Aldrich), caring 10 mM glucose (#103577-100, Agilent Technologies), 2mM glutamine (#103579-100, Agilent Technologies) and 10nM HEPES buffer (#P05-01100, PAN Biotech) with a pH of 7.4. 60 min later a standardized Seahorse XF Cell Mito Stress Test was run, sticking to the instructions given by the manufacturer: HUVECs were subsequently treated with Oligomycin, FCCP and Rotenone + Antimycin A.

### Quantitative profiling of metabolites by liquid chromatography and mass spectrometry (LC-MS/MS)

Approximately 1.2 million HUVECs were seeded onto a 10 cm diameter petri dish (#353003, Corning) with 5 ml of EBM with 2% FBS and left for 9 h to let cells attach to the surface followed by medium change and cytokine stimulation. Cells were harvested after 6 h, 12 h, 18–36 h, with four independent biological replicates per timepoint to avoid batch effects. All cells were harvested, washed with phosphate buffered saline (PBS), centrifuged, flash frozen as cell pellets and stored at −80 °C.

Cell pellets were thawed and homogenised with 0.2 mL of ice-cold PBS supplemented with 0.1% of protease inhibitor cocktail (Sigma, P8340), using a cordless pestle motor and disposable pellet mixers (VWR Nr. 47747-366). Whole cell lysates were aliquoted for downstream measurement of total protein concentration, flash-frozen and stored at -80 degrees Celsius. Sulfur-containing metabolites as well as creatinine, S-adenosylmethionine and S-adenosylhomocysteine were determined according to a previously published procedure [32]. Folates were determined under chromatographic conditions described in prior work, with modifications [32]. Briefly, stock solutions of folates were supplemented with ascorbic acid (10 mM). Calibration curves for all metabolites were generated from individual stock solutions prepared in house. Amino acids and neurotransmitters were determined as described in prior work [32]. Quantitation accuracy was examined by monitoring homocysteine concentrations in an external quality control in serum provided by the European Research Network for the evaluation and improvement of screening, diagnosis, and treatment of Inherited disorders of Metabolism (ERNDIM IQCS, SAS-02.1 and SAS-02.2 from MCA Laboratories, Winterswijk, Netherlands). For all other metabolites, quantitation trueness was tested by examining metabolite concentrations in plasma from a previously validated sample isolated from an adult healthy control individual with respect to standard reference ranges, using the same calibration curves and LC-MS/MS running conditions. Intracellular and extracellular metabolite concentrations were normalized by total protein concentration of the cell lysate and expressed as nmol metabolite/mg protein. Total protein concentration was determined using the bicinchoninic acid assay (Thermofisher Nr. 23225).

### Signal processing and data analysis

Quantification of metabolites was carried out with Analyst^®^ 1.7.2 software, 2022 AB Sciex. The resulting data were then analyzed and visualized using the publicly available Metaboanalyst software or using R Studio software (R Version 4.3) to perform principal component analysis. Metaboanalyst proceeded data were auto scaled and therefore connected data were normalized to the mean of the referred control group (EBM) (Fig. 5A). Other univariate data were analyzed using absolute concentrations to evaluate magnitude.

### Analysis and visualization

Datasets were checked for gaussian distribution using the Shapiro-Wilk test. Nonparametric data analysis was conducted calculating p-values with the Mann-Whitney or Kruskal-Wallis test. Normal distributed data was analyzed using unpaired t-test or analysis of variance (ANOVA). In multi group datasets, comparisons were run against all groups. Multiple comparison adjustments were performed with the Benjamin, Krieger and Yekutieli (BKY) method where applicable. Statistically significant events were determined by *p*-values < 0.05 and marked with an asterisk. Events with p-values > 0.05 were titled with ns (non-significant). Fold changes (FC) and group differences (∆) were calculated by using the median for nonparametric data and the mean for parametric data.

Either Violin plots showing medians and quartiles or bar charts showing medians and interquartile range were used for nonparametric data visualization. Bar charts showing means and standard deviation (SD), or line charts showing means and standard error of the mean (SEM) were used for parametric data. Graphs were created with Prism software (Version 10) or R Studio software (R Version 4.3).

## Results

### Elevated vitreous levels of IL-6 and sIL-6R in patients with diabetic retinopathy

**Fig. 1:**
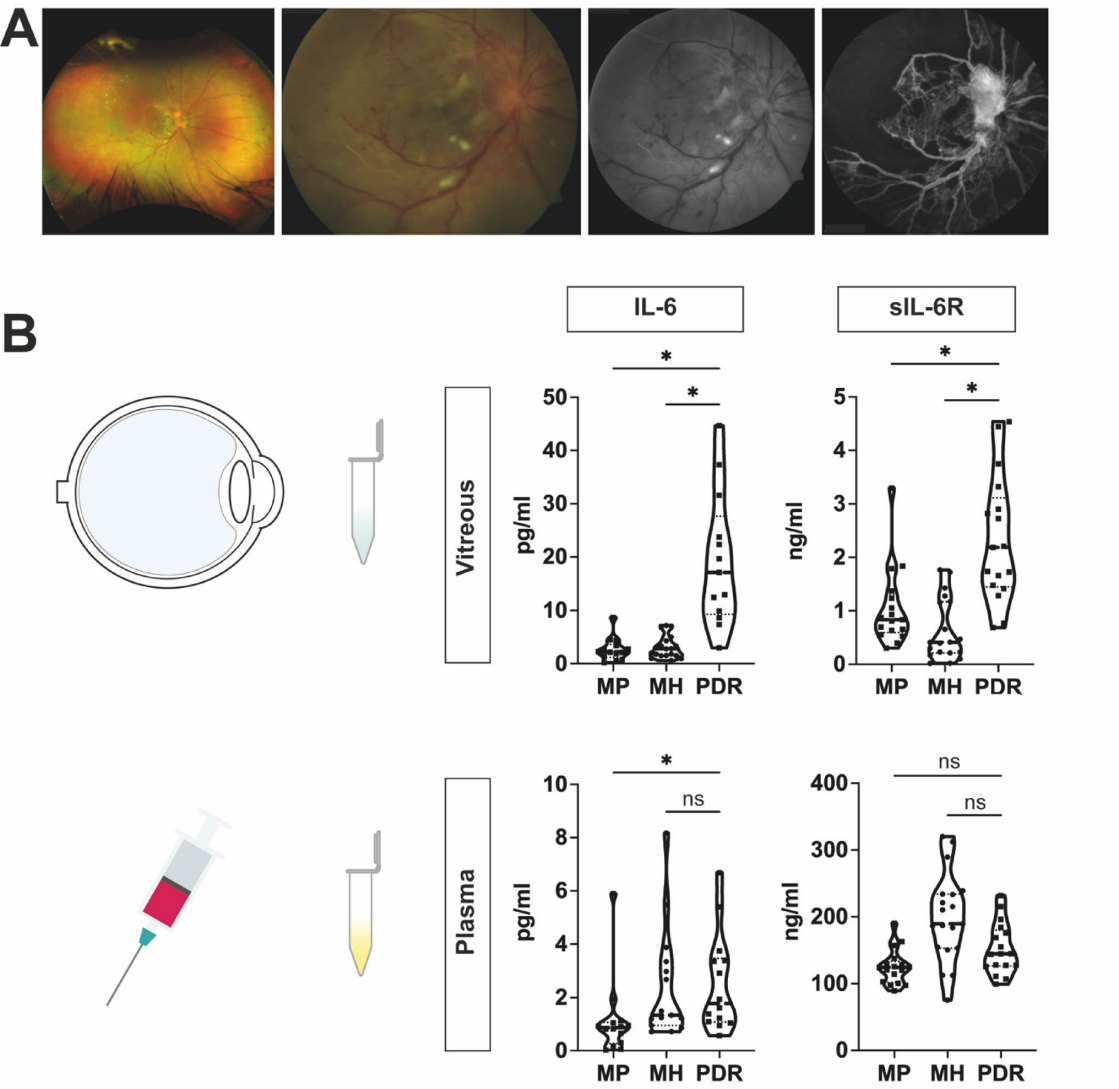
Elevated vitreous levels of IL-6 and sIL-6R in patients with proliferative diabetic retinopathy. **A** Fundus images and fluorescein angiography of a patient with retinal ischemia and proliferative diabetic retinopathy (PDR). **B** Protein levels of IL-6 and sIL-6R in vitreous and plasma samples from patients with PDR, macular pucker (MP) and macular hole (MH). Kruskal-Wallis, n (IL-6) = 13–19, n (sIL-6R) = 17–19, * = *p* < 0.05 ns = non-significant

To investigate whether patients with proliferative diabetic retinopathy (PDR) (Fig. 1A) show altered levels of interleukin-6 (IL-6) and its soluble IL-6 receptor (sIL-6R), vitreous humor and plasma samples from 17 PDR patients were compared to 17 patients with macular pucker (MP) and 19 patients with macular hole (MH), both serving as control groups (Fig. 1B) (Table 1).

**Table 1:** Study population age and sex matched, control groups without diabetes

	PDR	MP	MH
Number of patients	17	17	19
Median Age (IQR)	70 (13)	69 (8)	68 (10)
Sex (n female, %)	7 (41%)	8 (47%)	12 (63%)

IL-6 levels were significantly elevated in the vitreous of PDR patients compared to clinical control groups such as patients with MP (FC = 7.8) and MH (FC = 6,1). Results for plasma samples were less consistent with significant difference in IL-6 levels between PDR and MP patients (FC = 2.0), while the difference between PDR and MH patients (FC = 1.4) was not significant. Looking at absolute values, the IL-6 concentration in the vitreous was higher than in the plasma (PDR-median: 17.1 pg/ml in vitreous vs. 1.8 pg/ml in blood, MP-median: 2.2 pg/ml in vitreous vs. 0.9 pg/ml in blood and MH-median: 2.8 pg/ml in vitreous vs. 1.3 pg/ml in blood). The vitreous levels of sIL-6R were significantly increased in PDR compared to patients with MP (FC = 2.8) and MH (FC = 5.5). Plasma levels of sIL-6R were not significantly altered comparing PDR patients to the clinical control groups (MP and MH). Overall sIL-6R concentrations were higher than IL-6 levels in the vitreous as well as in the plasma, while plasma levels exceeded vitreous levels (PDR-median: 2.2 ng/ml in vitreous vs. 144.6 ng/ml in blood, MP-median: 0.8 ng/ml in vitreous vs. 124.3 ng/ml in blood and MH-median: 0.4 ng/ml in vitreous vs. 189.7 ng/ml in blood.) (Fig. 1C) (Table 2).

**Table 2:** Elevated vitreous levels of IL-6 and sIL-6R in patients with proliferative diabetic retinopathy

	IL-6 (pg/ml)			sIL-6R (ng/ml)		
	MP	MH	PDR	MP	MH	PDR
Vitreous (IQR)	2.2 (2.5)	2.8 (2.8)	17.1(18.4)	0.8 (0.7)	0.4 (1.0)	2.2 (1.7)
Plasma (IQR)	0.9 (0.8)	1.3 (2.4)	1.8 (2.4)	124.3 (31.8)	189.7 (81.1)	144.6 (53.4)

Vitreous and plasma samples from patients with diabetic retinopathy (PDR) and macular pucker (MP) or macular hole (MH) as clinical control groups. Median with IQR (interquartile range)

### IL-6 trans-signaling activates a broad spectrum of downstream signals whereas IL-6 cis-signaling is restricted to STAT3 activation in angiogenic signaling

**Fig. 2:**
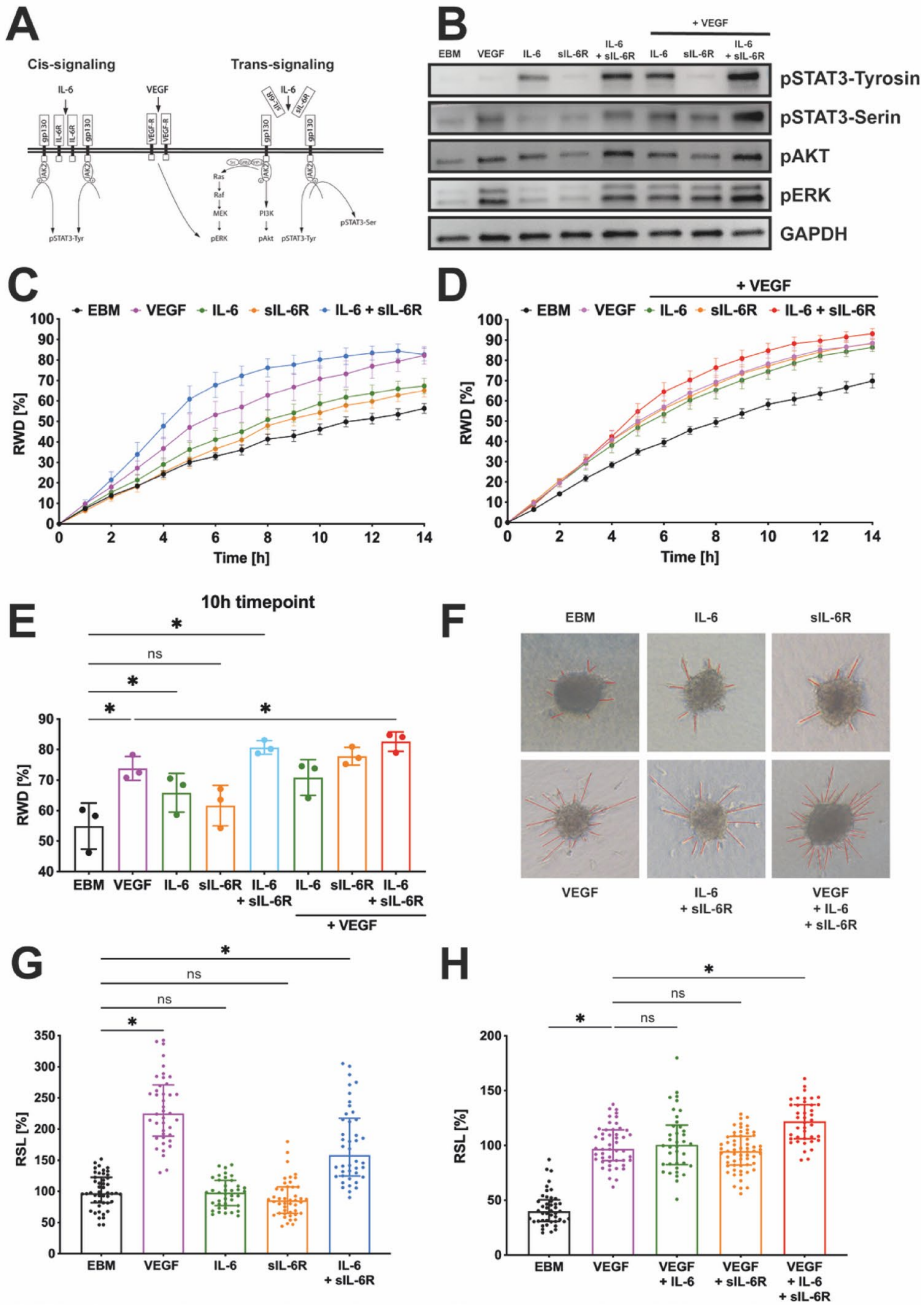
IL-6 trans-signaling exerts a pro-angiogenic effect complimentary to VEGF-induced angiogenesis. **A** Pathway of IL-6 cis- and trans-signaling. **B** Phosphorylation state of IL-6-signaling in HUVECs grown in EBM and treated for 15 min with combinations of VEGF, IL-6 and sIL-6R, Western Blot analysis. **C**–**E** Wound scratch assay measuring the migratory effect of EBM, VEGF, IL-6, sIL-6R, IL-6 + sIL-6R and VEGF co-stimulation on HUVECs. **C**, **D** Representative time course over 14 h. Relative wound density (RWD) without **C** or with **D** VEGF co-stimulation, graph showing the mean ± SEM, *n* = 6–8 technical replicates. **E** Statistical analysis of the RWD at 10 h, ordinary one-way ANOVA, *n* = 3 independent biological experiments, * = *p* < 0.05, ns = non-significant, error bar = SD. **F** Representative images from the spheroid sprouting assay. Relative sprouting length (RSL) of **G** EBM, VEGF, IL-6, sIL-6R, IL-6 + sIL-6R treatment and **H** VEGF co-stimulation on HUVECs, Kruskall-Wallis, *n* = 3 independent biological experiments with minimum 12 spheroids per group, * = *p* < 0.05, ns = non-significant, error bar = SD

IL-6 can either signal via its membrane bound IL-6 receptor and gp130 co-receptor known as cis-signaling or bind sIL-6R extracellularly and then interact with the gp130 co-receptor to perform trans-signaling (Fig. 2A). Western blot analysis of HUVECs showed that co-treatment with IL-6 + sIL-6R activated a broader spectrum of signaling pathways associated with angiogenesis (pSTAT3-Tyr, pSTAT3-Ser, pAKT, pERK) than treatment with IL-6 alone (pSTAT3-Tyr). Adding VEGF seemed to enhance IL-6 + sIL-6R induced ERK phosphorylation (Fig. 2B) (Semi-quantitative analysis: Sup. 1).

### IL-6 trans-signaling has a proangiogenic effect on vascular endothelial cells

IL-6 trans-signaling (IL-6 + sIL-6R) induces a pro-migratory effect on HUVECs similar to that of VEGF in a 2D scratch wound assay, monitoring wound density over time (Fig. 2C). Co-stimulation (VEGF + IL-6 + sIL-6R) only leads to a minor increase in relative wound density (RWD) compared to VEGF (Fig. 2D). These observations can be quantified by analyzing the 10 h post treatment timepoint (Fig. 2E). VEGF showed its angiogenic potential by increased RWD compared to the EBM (control) group (∆ = 18.9% points). IL-6 trans-signaling had a significantly higher RWD than EBM (∆ = 25.8% points). This was also observed for IL-6 treatment, although with a markedly reduced magnitude (∆ = 10.9% points). VEGF + IL-6 + sIL-6R co-stimulation showed slight but significantly elevated RWD compared to VEGF only treatment (∆ = 8.8% points).

To further validate these findings, we examined them in a 3D model [33]. The spheroid sprouting assay revealed enhanced endothelial cell sprouting (RSL) in particular with IL-6 trans-signaling (∆ = +61.4% points) compared to the basal sprouting rate (EBM control group), with VEGF stimulation surmounting its effect (∆ = +128.1% points) (Fig. 2G + H). The 3D sprouting was not fully saturated by exclusive VEGF treatment, as additional IL-6 + sIL-6R treatment induced a significantly enhanced effect (VEGF + IL-6 + sIL-6R vs. VEGF, ∆ = +25.1% points) (Fig. 2H). Singular IL-6 (cis-signaling) or sIL-6R stimulation, with or without VEGF co-stimulation, failed to show significant functional effects and remained at levels comparable to the respective baseline control. The angiogenic potential of IL-6 trans-signaling was cross-validated with human retinal microvascular endothelial cells (HRMVECs) also using the spheroid sprouting and migration assay, showing effects comparable to those observed in HUVECs (Sup. 2).

### IL-6 trans-signaling degrades barrier functions of vascular endothelial cells

**Fig. 3:**
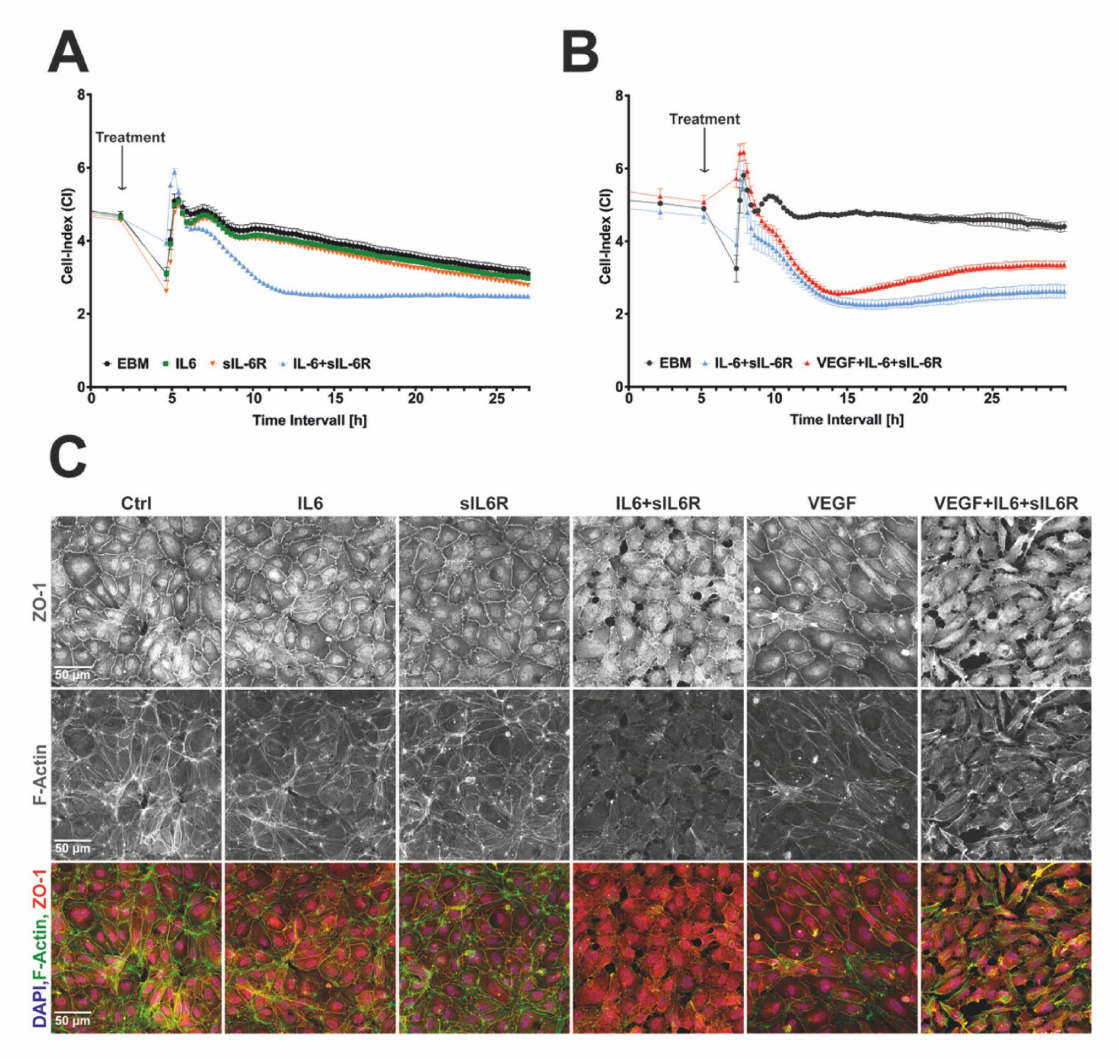
IL-6 trans-signaling impairs the blood retinal barrier in vitro. Impedance of HUVEC monolayers over time in response to treatment with **A** EBM-control, IL-6, sIL-6R and IL-6 + sIL-6R and **B **EBM-Control, VEGF, IL-6 + sIL-6R and VEGF + IL-6 + sIL-6R. Graphs depict the mean ± SEM. **C** Immunocytochemistry for ZO-1, F-Actin and DAPI (overlay) of HUVECs under the mentioned conditions for 12 h. Each sample is representative for 3 independent biological replicates

Since the integrity of the blood retinal barrier is a key aspect of retinal vascular disease, we examined the effect of IL-6 cis- and trans-signaling on the barrier function of vascular endothelial cells. Impedance measurements of HUVEC monolayers were performed as in vitro retinal blood barrier model. Endothelial cell monolayers form an electrical and physical barrier, generating a constant electrical resistance, which can be reported as a „Cell Index“. A reduced barrier function is associated with decreasing values. HUVECs treated with IL-6 + sIL-6R showed a significant time-dependent decrease in impedance with an absolute minimum after 8–12 h. Treatment with IL-6 or sIL-6R alone had no effect on impedance (Fig. 3A). Treatment with VEGF alone slightly increased impedance whereas a combination of IL-6 + sIL-6R with VEGF induced a significant loss of impedance (Fig. 3B). These functional effects correlated with morphological changes as visualized by immunocytochemistry. IL-6 + sIL-6R induced a reduction and a change in the localization of the cell adhesion marker ZO-1, a reduction of F-actin fibers and loss in cell-cell contacts. In combination with VEGF, this effect was even more pronounced. In contrast, IL-6, sIL-6R or VEGF alone had no effect on morphology, expression or localization of ZO-1, or F-Actin (Fig. 3C + D). To further evaluate cell adhesion and barrier function in our conditions we also looked at the tight junction marker occludin. Despite the relatively weak overall signal, a clear reduction in the junctional localization of occluding was evident in the IL-6 + sIL-6R and VEGF + IL-6 + sIL-6R treatment groups while all other stimulation groups (VEGF, IL-6, sIL-6R) did not reveal any clear alterations (Sup. 3D).

### IL-6 trans-signaling shifts metabolic activity to higher states in HUVECs

**Fig. 4:**
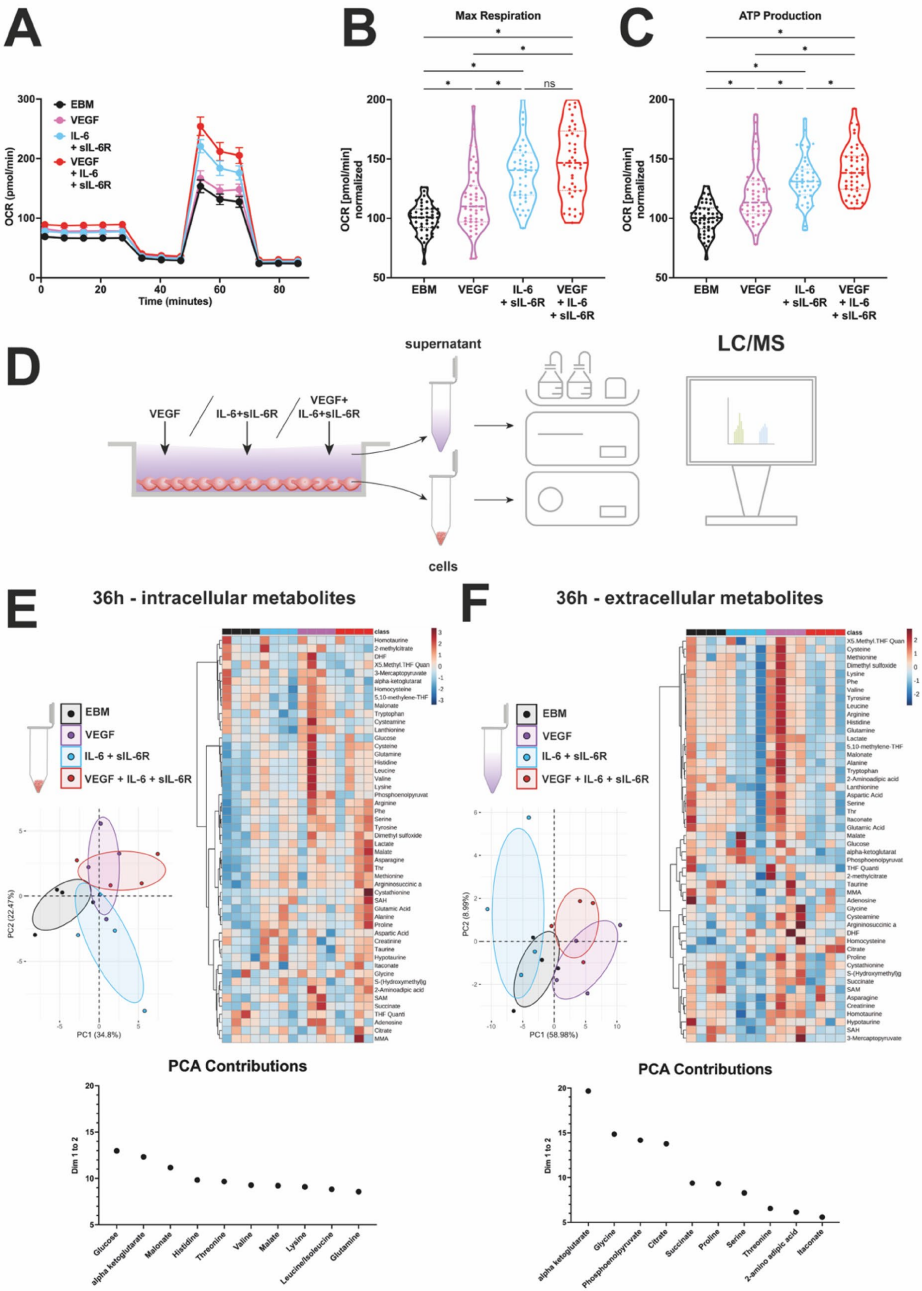
IL-6 trans-signaling induces a metabolomic shift in vascular endothelial cells. **A** Seahorse Flux analysis of HUVECs exposed to EBM, VEGF, IL-6 + sIL-6R and VEGF + IL-6 + sIL-6R for 15 h, representative graph showing the mean ± SEM, *n* = 6–8. **B** Quantitative analysis of maximum respiration **C** and ATP production. Kruskal Wallis test, *n* = 6 independent experiments, * = *p* < 0.05, ns = non-significant. (**D** Schema of sample collection for targeted metabolomics via LC/MS. **E**, **F** Principal Component Analysis (PCA), top 15 contributing metabolites considering Dim1 + Dim2 and heatmap visualizing changes between four treatment groups for intracellular **E** and extracellular **F** metabolites. *n* = 4 independent experiments per group

Changes in the metabolic activity of cells represent an important requirement for enhanced angiogenesis under hypoxic conditions with increased VEGF levels. Therefore, we next aimed to elucidate the effect of IL-6 trans-signaling on vascular endothelial cell metabolism. We used a Seahorse assay to analyze changes in oxygen consumption rate (OCR) of vascular endothelial cells in EBM treated with VEGF, IL-6 + sIL-6R or VEGF + IL-6 + sIL-6R. Following FCCP administration leading to maximum OCR in HUVECs, the IL-6 + sIL-6R and VEGF + IL-6 + sIL-6R groups rose to higher OCR levels than the EBM (control group). Interestingly IL-6 trans-signaling exceeded VEGF in maximum OCR levels. With the latter showing slightly higher OCR levels than EBM (Fig. 4A). Extracting defined quantitative bioenergetic parameters allows objective evaluation and significance testing. IL-6 + sIL-6R significantly increased maximum respiration and ATP-production compared to the EBM group. VEGF on its own also elevated these parameters, notably these effects were significantly weaker compared to IL-6 + sIL-6R treatment. Co-stimulation of VEGF and IL-6 + sIL-6R lead to further elevated ATP production but no increase in maximum respiration compared to VEGF alone (Fig. 4B, C).

### Metabolomics reveal different profiles for IL-6 trans-signaling- and VEGF-induced metabolism and give hints to diverging energy supply pathways

**Fig. 5:**
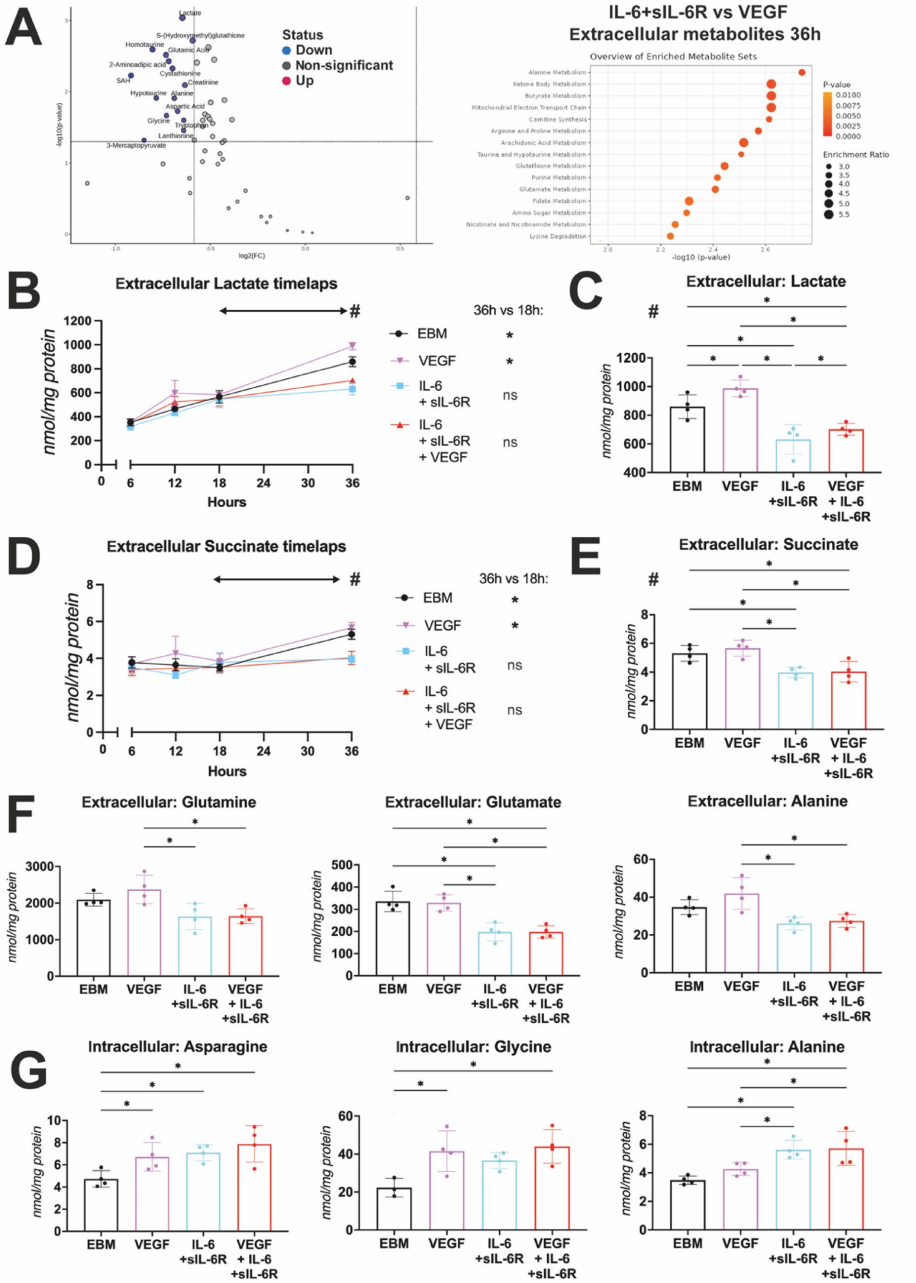
**IL-6 trans-signaling alters sugars and amino acids in central energy supply pathways while VEGF increases lactate accumulation linked to enhanced glycolysis.** **A** Metaboanalyst metabolite set enrichment analysis for extracellular IL-6 + sIL-6R vs. VEGF, Top 15 altered pathways and volcano plot highlighting metabolite comparisons with unpaired t-test with alpha < 0.05 and log2(FC) > 1.5. **B** Extracellular lactate timelaps (6–36 h post stimulation), graph visualizing mean ± SEM, statistical analysis of timelaps between 18 h and 36 h for EBM control and all treatment groups, unpaired t-test, *n* = 4, * = *p* < 0.05. **C** Extracellular lactate concentrations 36 h post stimulation, ordinary one-way ANOVA, *n* = 4, comparing all sets to each other only showing * = *p* < 0.05, error bar = SD. **D** Extracellular succinate timelaps (6–36 h post stimulation), graph visualizing mean ± SEM, statistical analysis of timelaps between 18 h and 36 h for all treatment groups, unpaired t-test, *n* = 4, * = *p* < 0.05. **E** Extracellular succinate concentrations 36 h post stimulation, ordinary one-way ANOVA, *n* = 4, comparing all sets to each other only showing * = *p* < 0.05, error bar = SD. **F** Extracellular metabolites visualizing amino acid (glutamine, glutamate and alanine) concentrations 36 h post stimulation, ordinary one-way ANOVA, *n* = 4, comparing all sets to each other only showing * = *p* < 0.05, error bar = SD. **G** Intracellular metabolites visualizing amino acid (asparagine, glycine, alanine) concentrations 36 h post stimulation, ordinary one-way ANOVA, *n* = 3–4, comparing all sets to each other only showing * = *p* < 0.05, error bar = SD, one outlier detected and left out for glycine with IQR*1.5 method

Targeted metabolomics was applied via LC-MS/MS to track changes in the concentrations of 50 metabolites covering glycolysis, tricarboxylic acid cycle (TCA) intermediates and amino acid metabolism over time in the supernatants (extracellular metabolites) and cells (intracellular metabolites) in response to cytokine treatment (Fig. 4D). Principal component analysis (PCA) revealed clustering for all groups regarding intracellular (Fig. 4E) and extracellular metabolites (Fig. 4F). This clustering was time dependent with the greatest divergence occurring 36 h after stimulation (Sup. 4 A + B). Therefore, the 36 h timepoint was selected for subsequent comparison of the metabolic profiles among treatment groups. The biggest difference in clustering was observed in the extracellular compartment between VEGF and IL-6 + sIL-6R treatment groups (Fig. 4F). This is especially interesting as VEGF and IL-6 + sIL-6R showed different effects on the endothelial barrier and varying magnitudes of response in the Seahorse analysis, in which VEGF exhibited an inferior capacity in inducing maximal respiration compared to IL-6 + sIL-6R. Comparing VEGF and IL-6 trans-signaling, 14 metabolites were significantly decreased in the extracellular compartment of IL-6 + sIL-6R treated HUVECs. Notably Lactate, a glycolytic end product, was among the decreased metabolites. Top 5 modified metabolite pathways identified by Metaboanalyst were alanine metabolism, ketone body metabolism, butyrate metabolism, mitochondrial electron transport chain (ETC) and carnitine synthesis. Important to note is that the ketone body metabolism, butyrate metabolism and mitochondrial electron chain transport all have succinate as their sole referred metabolite in the pathway calculation (Fig. 5A). In summary, we observed major alterations in lactate levels and modifications of the mitochondrial electron transport chain linked to succinate concentrations. This raises the possibility that VEGF and IL-6 + sIL-6R could induce different mechanisms to secure energy supply. The IL-6 + sIL-6R treated groups showed a time-dependent increase similar to the other groups, but lactate increase leveled off after 18 h, reaching a plateau. In contrast, extracellular lactate accumulation in the EBM and VEGF groups increased significantly between 18 and 36 h post-stimulation (Fig. 5B). Zeroing in on the 36-hour timepoint we observed that the extracellular lactate levels in the VEGF-treated group were significantly higher than in all other groups. IL-6 + sIL-6R treatment generated the lowest lactate concentration, consequently exhibiting the greatest difference from VEGF (∆ = -356.7 nmol/mg protein). Co-stimulation of IL-6 + sIL-6R and VEGF resulted in a significant reduction of lactate levels compared to VEGF treatment alone (∆ = -286.0 nmol/mg protein) (Fig. 5C). These findings suggest that IL-6 trans-signaling and VEGF exert opposing effects on lactate metabolism, indicating potential antagonistic roles in glycolytic control. Intracellular lactate levels did not increase over time, as lactate accumulation would lead to acidosis and thus most lactate is actively exported into the extracellular compartment via monocarboxylate transporters (MCT) [34, 35] (Sup. 4D).

Succinate, a metabolic intermediate of the TCA cycle that also fueled the ETC at complex II via succinate dehydrogenase (SDH), accumulated extracellularly in EBM- and VEGF-treated groups over time. In contrast, IL-6 + sIL-6R stimulated cells did not show accumulation of succinate and had steady extracellular concentrations (Fig. 5D) resulting in significantly lower levels in IL-6 + sIL-6R treated groups compared to either EBM or VEGF at 36 h post stimulation (Fig. 5E). These results further suggest that IL-6 + sIL-6R could induce HUVECs to obtain their energy supply in different ways than when treated with VEGF.

In addition to shifts in energy supply pathways, changes in amino acid profiles were also observed. For example, extracellular glutamate (glutamic acid) levels were highlighted among significantly lowered metabolites for IL-6 + sIL-6R compared to VEGF (Fig. 5A). Univariate data analysis comparing all treatment groups revealed that glutamate was significantly depleted in samples stimulated with IL-6 + sIL-6R compared to either EBM (∆ = − 138 nmol/mg protein) or VEGF (∆ = − 131 nmol/mg protein). A similar trend was observed for extra-cellular glutamine, an amino acid directly connected to glutamate and the most abundant amino acid in blood [36] and in our experimental setup. Concentrations again were significantly lower for IL-6 + sIL-6R compared to VEGF (∆ = − 741 nmol/mg protein) or EBM stimulation (∆ = − 461 nmol/mg protein), with the latter not being significant after correcting for multiple comparisons (Fig. 5F). Similar observations were made considering alanine. IL-6 + sIL-6R-induced levels were significantly below VEGF (∆ = − 15,91 nmol/mg protein) or EBM induced levels (∆ = − 8.67 nmol/mg protein), again the latter not being significant after correcting for multiple comparisons (Fig. 5F). Alanine metabolism exhibited the most significant alteration in the enrichment analysis when comparing VEGF to IL-6 + sIL-6R as stimuli. Additionally, it was one of only two amino acids, alongside asparagine, that showed a consistent and significant increase in intracellular levels across all IL-6 + sIL-6R- treated groups compared to EBM-treatment (Sup. 4 C). Interestingly, when looking at these metabolites, the intracellular compartment showed an inverse correlation compared to the extracellular compartment (Fig. 5F + G). IL-6 + sIL-6R stimulated cells significantly accumulated alanine compared to EBM-treatment (∆ = + 2.14 nmol/mg protein) (Fig. 5G). Besides altered alanine levels, glycine concentrations were significantly increased in groups containing VEGF, whereas exclusive IL-6 + sIL-6R treatment (∆ = + 19.26 nmol/mg protein) or VEGF + IL-6 + sIL-6R co-stimulation (∆ = + 20.70 nmol/mg protein) showed a similar increase (Fig. 5G). As mentioned earlier, asparagine levels showed elevated intracellular levels, with VEGF + IL-6 + sIL-6R displaying the highest levels (∆ = + 3.15 nmol/mg protein), followed by IL-6 + sIL-6R (∆ = + 2.38 nmol/mg protein) and VEGF leading to a modest increase (∆ = + 2.00 nmol/mg protein) when comparing to EBM.

### Targeted antibody treatment can reverse the effects induced by a combination of VEGF, IL-6 and sIL-6R

The observation that VEGF + IL-6 + sIL-6R impacted angiogenesis, endothelial barrier function and metabolism in HUVECs prompted us to assess the effects of complementary antibody treatment. While aflibercept binds VEGF as a decoy receptor of VEGFR1 and –R2, tocilizumab binds sIL-6R as well as membrane bound IL-6 receptor to block IL-6 signaling. Endothelial sprouting was significantly increased upon treatment with VEGF + IL-6 + sIL-6R compared to the basal sprouting rate (∆ = +130% points), indicating its strong promotion of angiogenic activity. Treatment with either aflibercept (∆ = –37% points) or tocilizumab (∆ = –20% points) significantly reduced VEGF + IL-6 + sIL-6R induced endothelial sprouting, but neither was sufficient to fully return sprouting to the basal level. In contrast, the combination of both antibodies completely abolished the pro-angiogenic effect of VEGF + IL-6 + sIL-6R, showing no significant difference to baseline sprouting (∆ = –123% points). These results suggest that simultaneously targeting multiple pathways can more effectively block pro-angiogenic signals in our experimental conditions.

**Fig. 6:**
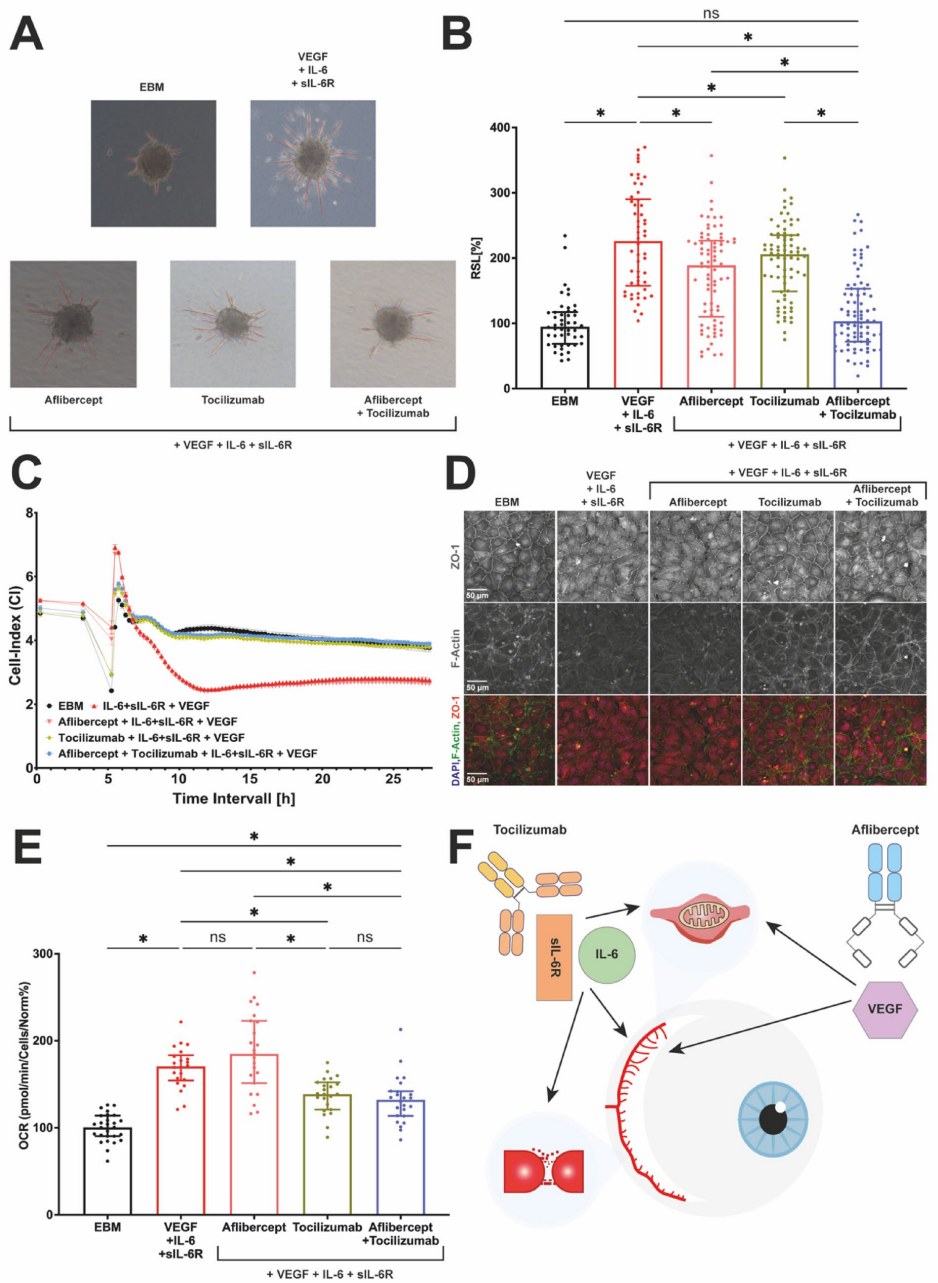
Targeted antibody treatment revokes VEGF + IL-6 + sIL-6R-induced effects. **A** Representative images **B** Quantitative results of the spheroid sprouting assay with HUVECs in response to stimulation with VEGF + IL-6 + sIL-6R, Aflibercept, Tocilizumab. Kruskal Wallis, *n* = 3 independent experiments with minimum of 12 spheroids per group, * = *p* < 0.05, error bar = SD. **C** Impedance of HUVEC monolayers over time in response to VEGF with IL-6 with sIL-6R in combination with Aflibercept or Tocilizumab or the combination of both antibodies. The data are expressed as the mean ± SEM. **D** Immunocytochemistry of ZO-1 (Zonula Occludens), F-Actin and DAPI (overlay) for HUVECs exposed to the above-mentioned conditions for 12 h. Each image is representative for 3 independent biological replicates. **E** Metabolic flux analysis of HUVECS treated with VEGF + IL-6 + sIL-6R, aflibercept, tocilizumab quantifying maximal respiration. Kruskal Wallis test, *n* = 3 independent assays with 6–8 replicates, * = *p* < 0.05, error bar = SD. **F** Graphical summary of VEGF and IL-6 + sIL-6R effects and aflibercept and tocilizumab inhibitions

Impedance measurements in HUVEC monolayers showed that tocilizumab with or without aflibercept significantly reduced the loss of function induced by IL-6 + sIL-6R + VEGF (Fig. 6C). In contrast, aflibercept had no significant effect in this setting (Fig. 6C). Supporting the functional measurements, tocilizumab and tocilizumab + aflibercept decreased changes in the expression and localization of ZO-1 and F-actin as well as the change in morphology as detected by immunofluorescence, in contrast to aflibercept alone (Fig. 6D). In the Seahorse assay we observed a significant reduction in oxygen consumption rate (OCR) when aflibercept and tocilizumab were added to cells treated with VEGF + IL-6 + sIL-6R. Aflibercept alone, however, could not reduce OCR. In contrast, treatment with tocilizumab decreased the VEGF + IL-6 + sIL-6 elevated OCR levels (Fig. 6E).

## Discussion

Our data strongly suggests that IL-6 trans-signaling (IL-6 + sIL-6R) drives loss of vascular integrity and retinal neovascularization with distinct but concordant metabolic alterations compared to VEGF leading to enhanced efficacy of combined anti-VEGF and anti-IL-6 therapy.

To underline the clinical relevance of our study, we first examined levels of IL-6 and its corresponding soluble receptor (sIL-6R) in the vitreous of patients with PDR, which were both elevated compared to the control groups (MP, MH) (Fig. 1B). Our study could hence confirm previously published data that reported higher vitreous levels for IL-6 in PDR [21–23]. In contrast to IL-6, only one early study tested for intravitreal sIL-6R concentrations, revealing increased levels in PDR [26], in line with our data, suggesting that IL-6 trans-signaling is currently understudied in the pathogenesis of retinal neovascular disease. Our study design comparing vitreous and blood IL-6/IL-6R levels could further highlight that these processes seem to develop on a local level, only affecting the vitreoretinal compound, as systemic blood cytokine levels are barely different between control groups and PDR patients (Fig. 1B). Elevated vitreous levels indicate that IL-6 and sIL-6R could play an important role in PDR, especially considering local intravitreal therapy according to anti-VEGF intravitreal drug administration. Missing systemic changes in IL-6 cytokine profile unfortunately do not provide sufficient evidence, that IL-6 or sIL-6R represent a promising biomarker for disease detection [23, 24].

Our study revealed that only IL-6 trans-signaling consistently promotes angiogenesis in vitro by enhancing cell migration and sprouting (Fig. 2). Contrary to previous reports, we show that IL-6 trans- signaling drives angiogenic features rather than impairing them [37]. These contradicting results could be explained by different IL-6 to sIL-6R concentration ratios. In our study, we used higher sIL-6R concentrations, as trans-signaling IL-6 + sIL-6R complexes rarely form - even with high cytokine concentrations [38]. Furthermore, sIL-6R is present at physiologically higher levels than IL-6 in the vitreous of PDR patients (Fig. 1) and using higher sIL-6R concentrations better mimics disease environment. To ensure the robustness of our observations, we employed the combination of 2D migration assays and 3D sprouting assays. As both assays capture different aspects of angiogenesis, and it has been suggested that 3D in vitro assays seem better suited to provide pathophysiological relevant results than 2D assays [33]. The observed results of our VEGF co-stimulation further support our claim that IL-6 trans-signaling acts as a pro-angiogenic pathway. The angiogenic response to combined VEGF + IL-6 + sIL-6R stimulation was not diminished relative to that induced by VEGF alone, indicating that IL-6 trans-signaling does not hinder angiogenesis in our experimental conditions. Moreover, IL-6 + sIL-6R + VEGF treatment exceeded the VEGF induced pro-angiogenic stimulus in both assays (Fig. 2E + H). The observed pro-angiogenic effect also aligns with our signaling studies that revealed activation of STAT3, ERK and AKT pathways by IL-6 trans-signaling in HUVECs (Fig. 2B), matching previously published data [39]. All three downstream pathways are closely connected to angiogenesis [40–42]. Taken together, all our data suggest that IL-6 trans-signaling functions as a pro-angiogenic inducer, acting in concordance with VEGF and contributing additively to the angiogenic stimulus. The observed additive effects display expected diminishing returns upon co-stimulation, thus highlighting the relevance of IL-6 trans-signaling as an angiogenic factor beyond VEGF.

We could also demonstrate that IL-6 trans-signaling results in reduced barrier function as indicated by decreased impedance values in HUVEC monolayers (Fig. 3A, B) aligning with observations made in other EC types [27]. Surprisingly, despite the common view of VEGF as barrier-disrupting factor in endothelial cells [43, 44], VEGF stimulated endothelial cells showed no alterations in impedance measurements. These discrepancies may be explained by the fact that, HUVECS, as venous endothelial cells, form a comparatively weaker barrier [45], which may limit detection of VEGF-induced permeability changes. VEGF responses are also concentration-dependent, with lower doses sometimes exerting stabilizing effects and higher doses typically enhancing permeability [46]. In our experimental setup, proliferative signaling of VEGF could be predominant and disguise barrier-related effects.

Metabolism is crucial in angiogenesis, as it fuels the formation of new blood vessels by providing energy and cellular building blocks like amino acids. We observed that HUVECs shift towards a more metabolically active phenotype upon IL-6 + sIL-6R stimulation, with higher maximum potential represented by increased OCR in maximum respiration and ATP production (Fig. 4A). We observed similar effects for VEGF, matching previously published data in HUVECs [47], but these were of a far lower magnitude compared to effects induced by IL-6 trans-signaling. Metabolomic profiling of metabolites and intermediates by LC-MS technology revealed previously uncharacterized insights and stimulus-dependent differences regarding energy supply pathways and amino acid profile in EBM-, VEGF- or IL-6 + sIL-6R-treated cells.

Lactate, which results from anaerobic glycolysis, is shuttled out of cells to prevent damage, and accumulates extracellularly [48]. VEGF induces glycolytic flux compared to EBM [49] which we could detect as elevated extracellular lactate concentrations. Reversely IL-6 + sIL-6R- treated cells showed decreased lactate levels compared to EBM (Fig. 5B, C). In the seahorse assay IL-6 + sIL-6R stimulation decisively surpasses VEGF-induced maximum respiration and oxygen-dependent ATP production (Fig. 4B, C). Taking both observations into account, we deliver first evidence that IL-6 trans-signaling and VEGF-signaling could differ in the energy supply mechanisms they trigger with the latter relying on anaerobic glycolysis which is well established, and IL-6 trans-signaling shifting towards OXPHOS and TCA for energy and metabolite supply [50]. The notion that VEGF and IL-6 + sIL-R act differently in the metabolic context is further underlined by the fact that aflibercept, in contrast to tocilizumab, is not capable to reduce OCR after VEGF + IL-6 + sIL-6R treatment. Furthermore, combined treatment with aflibercept and tocilizumab is not providing an enhanced effect on OCR regulation (Fig. 6E).

Our data further indicates that succinate accumulates extracellularly over time following VEGF-treatment, similar to the EBM control group. In contrast, sets containing IL-6 + sIL-6R show steady extracellular succinate levels (Fig. 5D, E). Succinate is an essential metabolite required in respiratory chain complex II of the mitochondrial ETC. Succinate dehydrogenase converts succinate to fumarate channeling electrons via flavine adenine dinucleotide (FAD) towards respiratory complex III [51]. Succinate accumulates when cells primarily depend on anaerobic glycolysis, which is well studied in cancer cells with disrupted oxidative phosphorylation [52]. Under normal circumstances succinate membrane permeability is limited, but acidification induced by lactate secretion can lead to succinate modification which excels extracellular transport via plasma membrane MCT1 even in the retina [53, 54]. This fits our correlating lactate and succinate observations considering VEGF stimulation (Fig. 5B, E) and a study describing reciprocal positive feedback between VEGF and succinate, with succinate levels falling upon VEGF inhibition [55]. Intriguingly the referred study observed elevated succinate levels in the vitreous of PDR patients [55]. Furthermore, succinate does not only function as a metabolite but also executes signaling functions. In macrophages LPS increases glutamine-derived succinate levels which stabilize HIF-α by blocking prolyl hydroxylase, which would overwise induce HIF-α degradation [56, 57]. SUCNR1 a G-protein-coupled receptor binding extracellular succinate [58] is capable of activating ERK in HUVECs and also inducing angiogenesis and VEGF secretion [59]. This study further revealed that succinate, SUCNR1 expression and VEGF are all upregulated in gestational diabetic placentas.

Regarding amino acids we observed extracellular depletion of glutamine and glutamate in groups treated with IL-6 + sIL-6R (Fig. 5F). Glutamine and glutamate are an important metabolic pathway for TCA anaplerosis and nucleotide synthesis leading to OCR increase [49]. This matches our data observed in the seahorse assay (Fig. 4A-C). Glutamine is the most abundant amino acid in circulating blood [60], relevant for angiogenesis and an essential carbon donor for the TCA cycle [61]. Under total glutamine depletion asparagine can rescue EC proliferation [62]. We observed higher intracellular asparagine concentrations (Fig. 5G) for all stimuli that were capable of inducing angiogenesis compared to EBM (Fig. 2). This underlines the critical role of asparagine in supporting cell proliferation, as it is targeted in cancer therapies using asparaginase [63].

The alanine metabolism pathway was enriched in all intracellular sets stimulated with IL-6 + sIL-6R (Sup. 4 C) and absolute intracellular alanine concentrations were elevated in all groups treated with IL-6 + sIL-6R (Fig. 5G). The metabolic role of alanine in ECs is still poorly understood. In the retina, cone photoreceptors may utilize the Cahill cycle, in which pyruvate is converted to alanine instead of reduction to lactate, while metabolizing glutamate to alpha-ketoglutarate [64].

Glycine was specifically upregulated in cells treated with VEGF (Fig. 5G). This finding aligns with earlier reports on VEGF stimulation that describe intracellular Glycine elevation, without detecting alterations for many other amino acids [47]. The study further observed that glycine can induce angiogenesis and promote increased OCR levels, also stating that glycine is essential for VEGF-induced angiogenesis. We did not observe any significant alterations in intracellular glycine with IL-6 + sIL-6R treatment, leading to the assumption that glycine could be an amino acid of essential importance to VEGF signaling. Interestingly, Glycine vitreous levels are elevated in patients with PDR [65].

Our metabolic data reveal pronounced differences between IL-6 trans-signaling and VEGF induced effects, pointing to mechanisms that may underline their independent actions.

Our study introduces a novel complementary treatment strategy combining aflibercept and tocilizumab to counteract the pathological effects induced by VEGF + IL-6 + sIL-6R. In spheroid sprouting assays, only the dual-antibody approach was able to completely abrogate angiogenic impulses, whereas monotherapy with either antibody produced only partial inhibition, leading to treatment insufficiencies (Fig. 6B). These findings highlight that targeting multiple axes simultaneously provides superior efficacy over single-agent interventions. Consistent with these findings, metabolic analyses revealed that VEGF + IL-6 + sIL-6R stimulation enhanced maximal OCR in HUVECs. Importantly, the combined antibody treatment achieved the most profound suppression of this metabolic shift (Fig. 6E). Furthermore, tocilizumab reversed VEGF + IL-6 + sIL-6R induced barrier defects confirmed by both impedance and morphological analyses (Fig. 6C, D). These observations raise the question if expanding present DR therapy protocols with IL-6 signaling inhibitors could be beneficial.

Tocilizumab is a broad inhibitor of IL-6 signaling, preventing both IL-6 cis- and trans-signaling. Our in vitro experiments suggest that IL-6 trans-signaling may be the dominant driver of endothelial dysfunction in this context. Employing a selective IL-6 trans signaling inhibitor, such as olamkicept [66], could therefore provide more precise mechanistic dissection and is particularly relevant for in vivo studies, given tocilizumab is ineffective in murine models, whereas olamkicept reliably blocks IL-6 trans-signaling in mice [67]. A deeper understanding of IL-6 signaling in retinal vascular diseases will be crucial to improve our therapeutic options for alleviating these common conditions.

## Supplementary Information

Below is the link to the electronic supplementary material.

Supplementary Material 1

## Data Availability

No datasets were generated or analysed during the current study.

## Abbreviations

AKT: Protein kinase B
CTL: Control group
DME: Diabetic macular edema
EBM: Endothelial basal medium
EC: Endothelial cell
EGM: Endothelial growth medium
ERK: Extracellular signal-regulated kinase
FBS: Fetal bovine serum
FC: Fold change
GAPDH: Glyceraldehyde-3-phosphate dehydrogenase
gp130: Glycoprotein 130
HUVEC: Human umbilical vascular endothelial cell
IL-6: Interleukin-6
IQR: Interquartile range
JAK: Janus kinase
LC/MS: Liquid chromatography–mass spectrometry
MCT: Monocarboxylate transporter
OCR: Oxygen consumption rate
OXPHOS: Oxidative phosphorylation
PBS: Phosphate buffered saline
(P)DR: (Proliferative) diabetic retinopathy
p: Phosphorylated
RSL: Relative sprouting length
RWD: Relative wound density
sIL-6R: Soluble interleukin-6 receptor
STAT: Signal transducer and activator protein
TCA: Tricarboxylic cycle
VEGF: Vascular endothelial growth factor
ZO: Zonula occludens
∆: Group differences
