# Supplementary material for: IL-6 trans-signaling: an overlooked driver of retinal neovascularization?
Source: Angiogenesis. 2025 Dec 19;29(1):11. doi: 10.1007/s10456-025-10022-8 (PMC12717152; doi:10.1007/s10456-025-10022-8)
Supplement: Supplementary file 1 — Supplementary Material 1 [file 10456_2025_10022_MOESM1_ESM.docx]

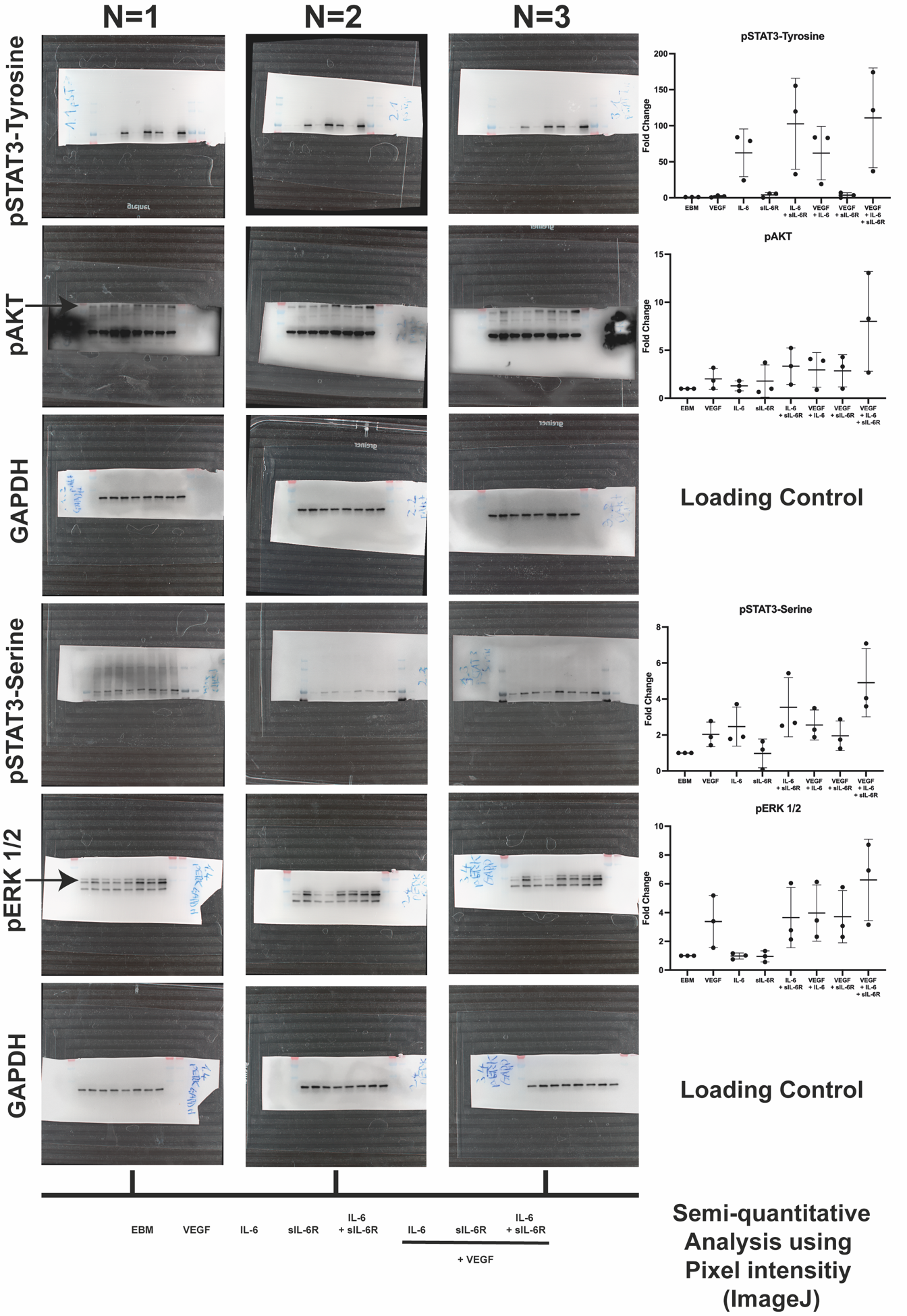


**Sup. 1 Semi-quantitative western blot analysis**

Total western Blot images of HUVECs grown in EBM and treated for 15min with combinations of VEGF, IL-6 and sIL-6R, from three different biological experiments. Semi-quantitative analysis of band intensity, using EBM control as reference for FC calculation.


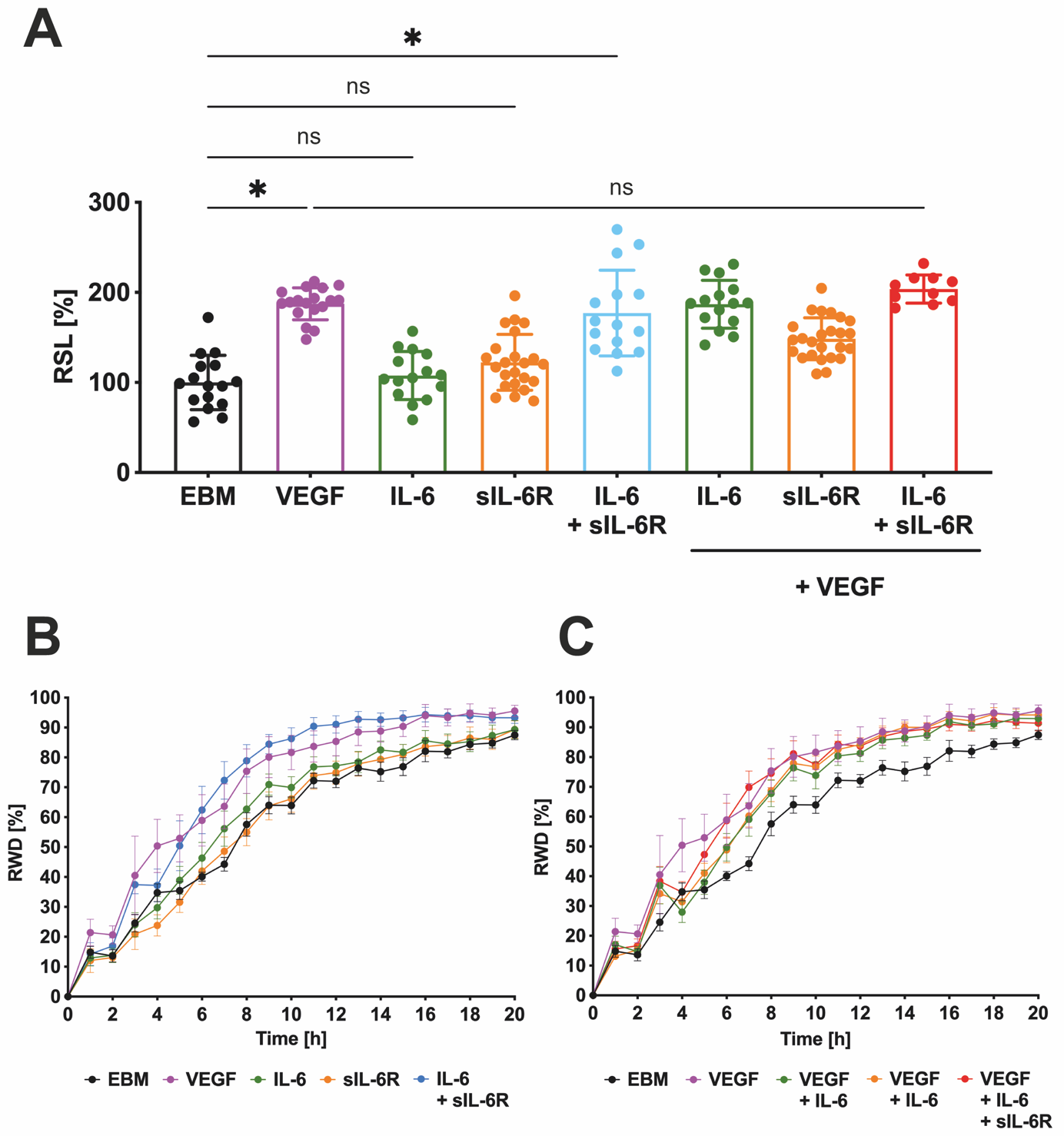


**Sup. 2 Cross-validation of IL-6 trans-signaling effects on angiogenesis in HRMVECs**

(A) Spheroid sprouting assay quantifying relative sprouting length (RSL) of EBM, IL-6, sIL-6R, IL-6+sIL-6R and VEGF co-stimulation on HRMVECs. Quantitative results of the RS. Kruskall-Wallis, n = 1 independent biological experiments with 10-23 spheroids per group, * = p < 0.05, ns = non-significant, error bar = SD. (B+C) Wound scratch assay measuring the migratory effect of EBM, VEGF, IL-6, sIL-6R, IL-6+sIL-6R and VEGF co-stimulation on HRMVECs. Representative time course over 20h. Relative wound density (RWD) without (B) or with (C) VEGF co-stimulation, graph showing the mean ± SEM, n= 6-8 technical replicates.


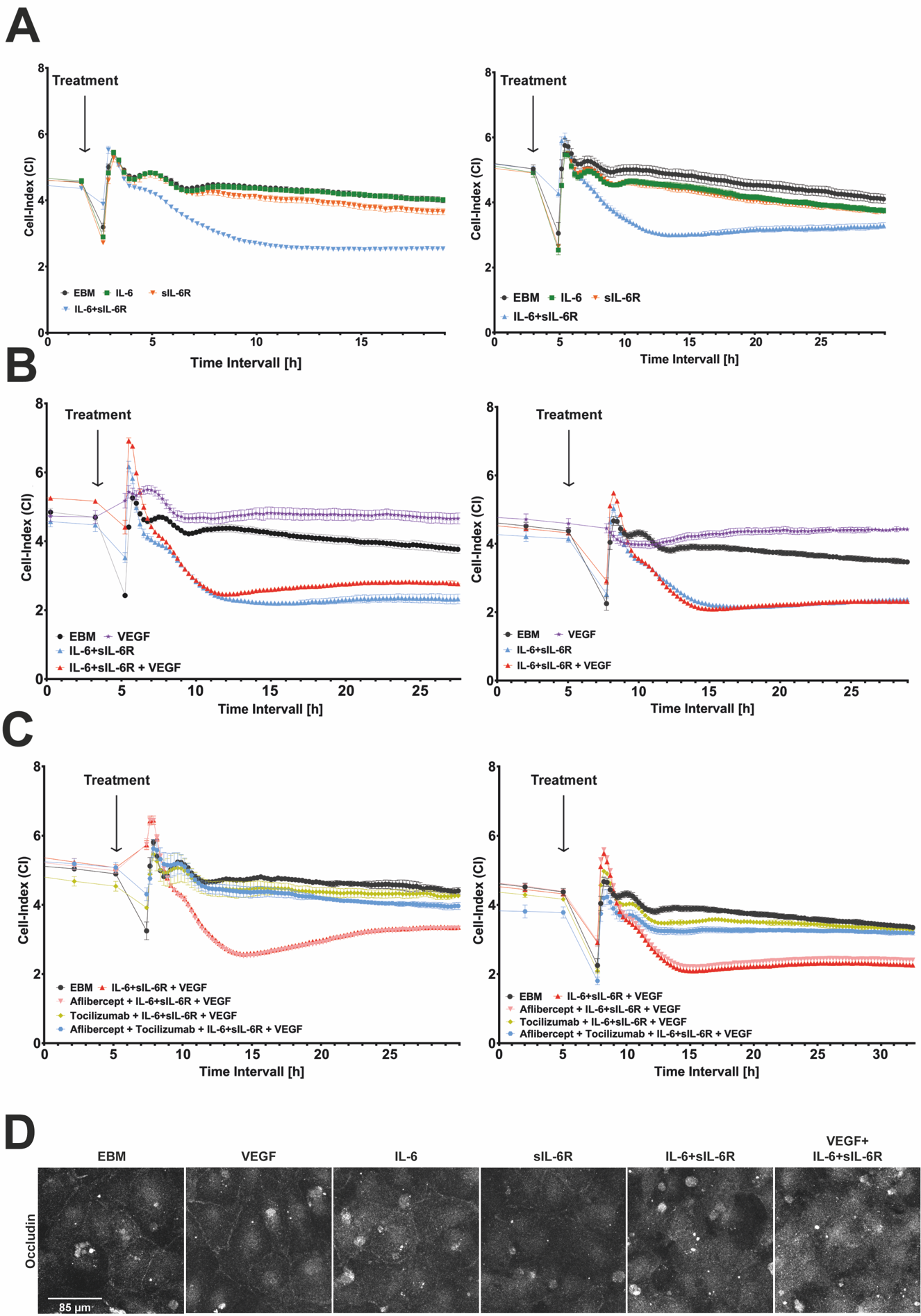


**Sup. 3 Impendence measurement images with RTCA XCelligence and occludin immunostaining**

Supplementary plots measuring impedance of HUVEC monolayers over time in response to treatment with

(A) IL-6, sIL-6R and IL-6+sIL-6R and (B) VEGF, IL-6+sIL-6R and VEGF+IL-6+sIL-6R. (C) Combinations of VEGF+IL-6+sIL-6R, Aflibercept and Tocilizumab. Graphs depict the mean ± standard deviation. (D) Occludin immunostaining of HUVECs after treatment at identical confluence with VEGF, IL-6, sIL-6R, IL-6+sIL-6R and VEGF+IL-6+sIL-6R for 12h. With EBM as experimental control group. n=1


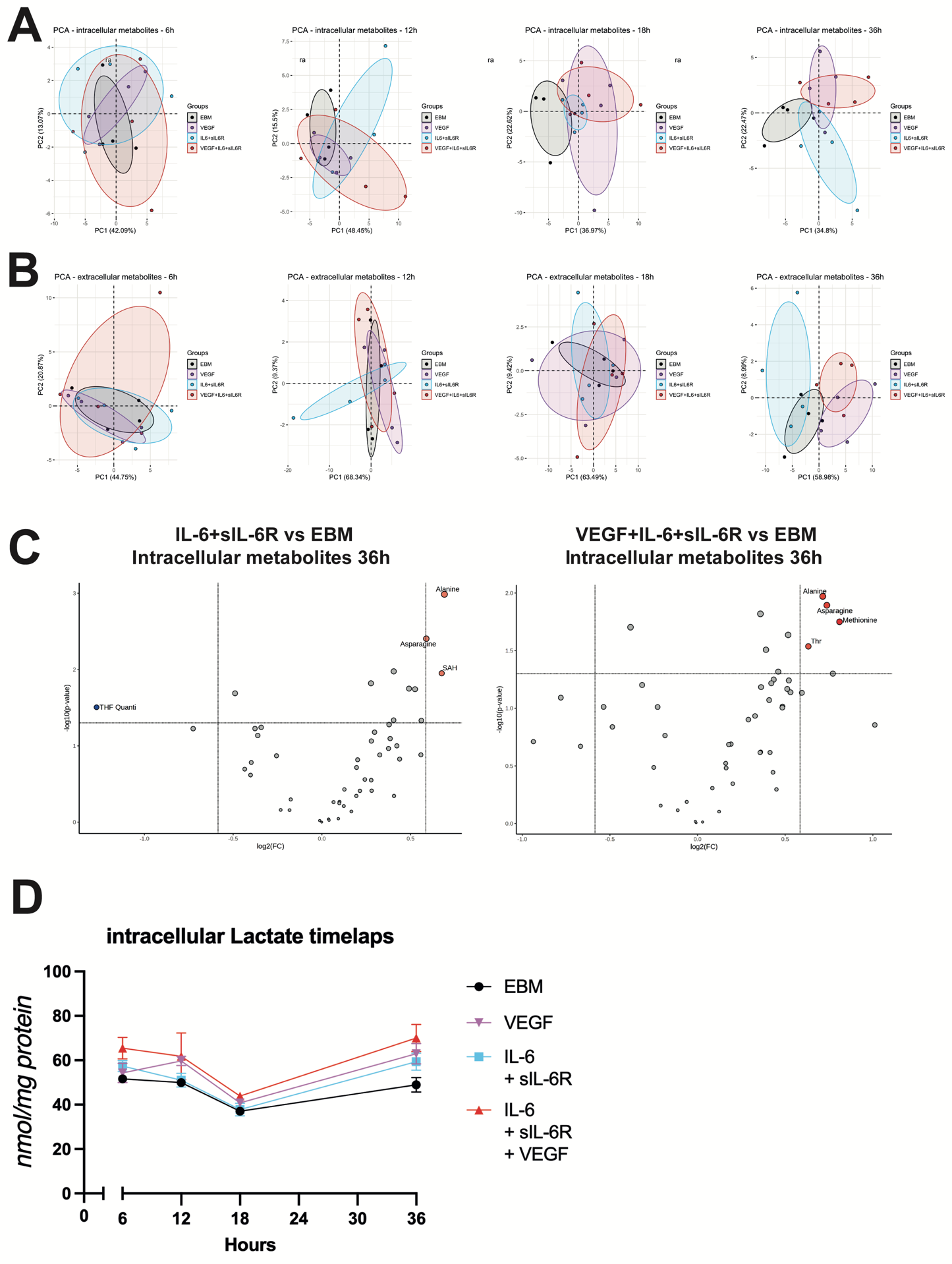


**Sup. 4 Metabolic profiling**

PCA for intra- (A) and extracellular (B) metabolic profiles of HUVECs treated with constellations of VEGF and IL-6+sIL-6R with timepoints of 6, 12, 18 and 36 hours. (C) Volcano plot highlighting intracellular metabolite comparisons with unpaired t-test with alpha < 0.05 and log2(FC) > 1.5, after 36 hours of stimulation. (D) Intracellular lactate timelaps (6h-36h post stimulation), graph visualizing mean ± SEM.
